# High-field superconductivity in C-doped MgB_2_ bulk samples prepared by a rapid synthesis route

**DOI:** 10.1038/s41598-020-74300-9

**Published:** 2020-10-19

**Authors:** D. Matera, M. Bonura, R. Černý, S. McKeown Walker, F. Buta, D. LeBoeuf, X. Chaud, E. Giannini, C. Senatore

**Affiliations:** 1 Department of Quantum Matter Physics (DQMP), University of Geneva, Geneva, Switzerland; 2 Laboratory of Advanced Technology (LTA), Geneva, Switzerland; 3 LNCMI, CNRS, EMFL, INSA Toulouse, Université Grenoble Alpes, Université Toulouse Paul Sabatier, Grenoble, France

**Keywords:** Superconducting properties and materials, Superconducting properties and materials, Applied physics

## Abstract

The upper critical field sets the thermodynamic limit to superconductivity. A big gap is present between the upper-critical-field values measured in MgB_2_ polycrystalline bulk superconductors and those of thin films, where values as high as ~ 50 T have been achieved at 4.2 K. Filling this gap would unlock the potential of MgB_2_ for magnet applications. This work presents the results of an extensive experimental campaign on MgB_2_ bulk samples, which has been guided by a Design of Experiment. We modeled the dependence of the upper critical field on the main synthesis parameters and established a new record (~ 35 T at 4.2 K) preparing C-doped bulk samples by a non-conventional rapid-synthesis route. This value appears to be an upper boundary for the upper critical field in bulk samples. Structural disorder in films seems to act selectively on one of the two bands where superconductivity in MgB_2_ takes place: this enhances the upper critical field while reducing the critical temperature only by few Kelvins. On the other hand, the critical temperature in bulk samples decreases monotonically when structural disorder increases, and this imposes a limit to the maximum achievable upper critical field.

## Introduction

The MgB_2_ superconductor has significant potential for practical applications. The main points of strength are its critical temperature close to 40 K, which may allow operating in cryogen-free environments, the low cost of precursor materials, and the ease of manufacture. However, today’s applications are limited to market niches, mainly constituted by low-field magnetic-resonance-imaging magnets and current leads^1–6^. The upper critical field, *H*_*C2*_, is well below 20 T at 4.2 K in polycrystalline binary MgB_2_, whilst it can exceed 50 T in carbon-doped films^7,8^. This value is about twice the *H*_*C2*_ of Nb_3_Sn, which is largely used in magnet applications and is considered one of the most promising candidates to realize next-generation particle-accelerator magnets^9^. In spite of the considerable efforts undertaken to reproduce the same results in polycrystalline bulk materials and wires, the highest *µ*_*0*_*H*_*C2*_ achieved so far is ~ 34 T at 4.2 K, as measured in a double-walled-carbon-nanotube (DWCNT) doped bulk sample^10^.

MgB_2_ has a planar structure with honeycomb B layers separated by Mg atoms. Strong sp^2^ hybrid σ bonding within the in-plane B atoms gives rise to the 2-dimensional σ band. Boron p_z_ orbitals lead to the 3-dimensional π band^11,12^. Superconductivity takes place on the two bands with different energy gaps of ~ 2.2 meV (π band) and ~ 7.0 meV (σ band) at 0 K^13^. Both superconducting gaps vanish at the bulk critical temperature *T*_*C*_^14^. Structural disorder can induce charge-carrier scattering on different channels: intraband scattering in each of the σ and π bands, and interband scattering between them^15^. Enhanced interband scattering leads to a decrease of *T*_*C*_, whilst theoretical models predict that *H*_*C2*_ can be significantly improved at low temperatures by selectively increasing the π-band intraband scattering^15,16^. Nanoscale disorder can be tuned by chemical doping^8,10,17^, irradiation^16,18^, and preparation conditions^18,19^. In the case of MgB_2_, C proved to be the most effective way to enhance *H*_*C2*_ by doping^10,20,21^. C is not expected to have the same effect on the MgB_2_ crystal structure in films and bulk samples^22,23^, and this may lead to variations in the scattering rates^19,22,24^. To date, the scenario that leads to record-high *H*_*C2*_ in films is unclear. The out-of-equilibrium environment typical of film-growth processing may play a key role in enhancing *H*_*C2*_. Indeed, *µ*_*0*_*H*_*C2*_(4.2 K) as high as ~ 44.5 T was measured in a binary film^25^, indicating that C doping is not an exclusive way to achieve very high *H*_*C2*_.

This work presents a systematic study of the effects of the synthesis conditions on *H*_*C2*_ for C-doped bulk samples. We employed a rapid-synthesis route, which allowed us to explore ranges of variation of the synthesis conditions not achievable with traditional techniques. By means of a Design of Experiment, we defined the *H*_*C2*_ response surface as a function of the main variables of the manufacturing route and thus determined the synthesis-parameter ranges that maximize *H*_*C2*_. We found that, in spite of an enhanced substitution rate of C in Mg(B_1−x_C_x_)_2_, *µ*_*0*_*H*_*C2*_ appears bounded to maximum values of ~ 26 T and ~ 35 T at 10 K and 4.2 K, respectively. These figures constitute new records for *H*_*C2*_ in bulk samples but remain far below what is achievable by the material in film form. We show that in bulk samples, *H*_*C2*_ and *T*_*C*_ correlate well with the lattice constant *a*. *H*_*C2*_ values of bulk samples (from this work and from the literature) on which disorder has been introduced by different sources can be estimated with an uncertainty below ~  ± 20% by knowing *T*_*C*_. The rapid-synthesis route allowed the production of samples with very-high irreversibility fields (*H*_*Irr*_). This is an important result for applications because *H*_*Irr*_ defines the maximum field at which superconductors can be operated in magnets. The potential scalability for large volume productions of wires and bulk materials is another point of strength of this technique.

## Results

The MgB_2_ bulk sample manufacturing process consists of a combination of the Internal Magnesium Diffusion (IMD) and the Powder-in-Closed-Tube (PiCT) techniques^26,27^. Samples prepared by IMD are typically characterized by high electrical connectivity^28^. The PiCT technique allows in turn achieving a high density of the reacted MgB_2_ phase and a high reproducibility of the sample properties^27^. Samples were reacted using a laboratory-made induction furnace, which allowed us to heat with ramp rates as high as ~ 1000 °C/min and to quench the reaction process by injecting high-pressure Ar on the sample crucible. We prepared few binary MgB_2_ samples as reference, and ~ 50 C-doped samples, most of them with a nominal composition Mg(B_0.9_C_0.1_)_2_. Indeed, x = 0.1 was proven to maximize *H*_*C2*_ in Mg(B_1−x_C_x_)_2_ polycrystalline samples in the case of DWCNT^10^, which is the C-dopant used in this work. Details about the manufacturing process are reported in section Methods.

Based on a previous work of ours^29^, we identified five synthesis parameters whose variation has a major effect on the samples’ superconducting properties, namely the heating ramp rate (*HR*), the dwell temperature (*T*_*d*_), the dwell time (*t*), the pressure of the Ar-gas quenching jet (*ArP*), and the pressure applied to the precursors before synthesis (*P*). These variables reciprocally interact in the determination of *H*_*C2*_, making the quest for the “best” synthesis conditions very challenging. The Design of Experiment (DoE) is a statistical tool used to determine the effects of experimental factors on a desired output in a system. It offers a set of advantages over the traditional one-variable-at-a-time approach since it can help to resolve parameter interactions and provide detailed maps of the system behavior^30^. We used two types of DoE in this work. First, we carried out a screening DoE with the aim of identifying the area of the synthesis-parameter space where the highest *H*_*C2*_ values are localized. We prepared 11 samples, which were characterized in terms of *T*_*C*_, *ΔT*_*C*_, *H*_*C2*_, *H*_*irr*_, and lattice parameters *a* and *c*. *H*_*C2*_ and *H*_*Irr*_ were evaluated at 10 K, for two main reasons. The first one is conceptual, as the key interest in MgB_2_ for magnet technology is for cryocooled systems operating above 4.2 K. The second one is practical and mainly related to the magnetic-field range (0–21 T) available at the University of Geneva, where most of the measurements were performed. The main properties of the samples prepared in the frame of the screening DoE are reported in Table 1. Based on this preliminary investigation, we performed a Response-Surface-Methodology (RSM) DoE, which is used to produce a detailed mathematical model of the process behavior as a function of the input variables^30,31^. The RSM DoE required the preparation of 26 samples, which were all investigated at the University of Geneva. A sample selection was further characterized up to 35 T in an extended temperature range down to ~ 2.5 K at the LNCMI (Grenoble, France). The superconducting and structural properties of the samples prepared in the frame of the RSM DoE are reported in Table 2. Details on both DoEs, including comments on the experimental reproducibility, are reported in Methods.

**Table 1:** Main properties of the 11 samples of the screening Design of Experiment: critical temperature *T*_*C*_, superconducting transition width *ΔT*_*C*_, irreversibility field *H*_*irr*_ at 10 K, upper critical field *H*_*C2*_ at 10 K, lattice parameters *a* and *c*. The estimated standard deviation for *a* and *c* is $\lesssim$ 0.0001 Å for all samples.

Sample ID	*T*_*c*_ (K)	*ΔT*_*c*_ (K)	*μ*_*0*_*H*_*irr*_* @*10 K (T)	*μ*_*0*_*H*_*C2*_* @*10 K (T)	*a* (Å)	*c* (Å)
SCR_1	37.8	0.6	17.7	21.5	3.0781	3.5206
SCR_2	31.4	1.9	18.4	22.9	3.0572	3.5213
SCR_3	–	–	–	–	–	–
SCR_4	37.6	0.5	17.4	20.7	3.0843	3.5248
SCR_5	–	–	–	–	–	–
SCR_6	37.2	1.2	17.9	22.8	3.0752	3.5204
SCR_7	33.6	3.0	13.9	20.3	3.0637	3.5235
SCR_8	32.6	2.5	16.8	20.5	3.0611	3.5242
SCR_9	34.8	1.4	13.4	17.5	3.0685	3.5216
SCR_10	32.3	2.5	16.3	20.3	3.0585	3.5221
SCR_11	37.1	1.3	16.9	22.6	3.0747	3.5263

**Table 2:** Main properties of the 26 samples of the Response-Surface-Methodology Design of Experiment: critical temperature *T*_*C*_, superconducting transition width *ΔT*_*C*_, irreversibility field *H*_*irr*_ at 10 K, upper critical field *H*_*C2*_ at 10 K, lattice parameters *a* and *c*. The estimated standard deviation for *a* and *c* is $\lesssim$ 0.0001 Å for all samples.

Sample ID	*T*_*c*_ (K)	*ΔT*_*c*_ (K)	*μ*_*0*_*H*_*irr*_* @*10 K (T)	*μ*_*0*_*H*_*C2*_* @*10 K (T)	*a *(Å)	*c* (Å)
RSM_1	33.9	3.4	18.0	23.6	3.0610	3.5267
RSM_2	35.8	1.8	17.3	20.2	3.0753	3.5257
RSM_3	33.4	3.2	13.9	21.4	3.0611	3.5264
RSM_4	32.9	3.7	16.0	22.8	3.0558	3.5217
RSM_5	36.2	1.2	17.8	22.8	3.0694	3.5203
RSM_6	29.0	2.0	13.7	20.5	3.0563	3.5261
RSM_7	33.7	6.6	17.3	23.1	3.0628	3.5255
RSM_8	28.8	4.3	13.4	20.9	3.0562	3.5270
RSM_9	36.2	2.3	18.0	21.9	3.0686	3.5263
RSM_10	34.4	2.1	15.2	22.9	3.0627	3.5256
RSM_11	34.1	3.8	16.7	24.4	3.0655	3.5245
RSM_12	30.3	2.7	17.2	23.8	3.0592	3.5244
RSM_13	29.2	8.3	14.0	21.1	3.0505	3.5244
RSM_14	30.4	4.1	14.2	20.3	3.0563	3.5255
RSM_15	32.2	2.3	18.8	23.9	3.0594	3.5235
RSM_16	36.3	0.6	18.6	22.5	3.0657	3.5256
RSM_17	35.0	2.5	18.1	24.0	3.0628	3.5268
RSM_18	31.8	4.4	16.8	23.7	3.0597	3.5263
RSM_19	32.8	3.0	15.9	23.7	3.0525	3.5191
RSM_20	27.4	1.9	11.9	17.3	3.0483	3.5190
RSM_21	26.8	4.1	7.8	17.1	3.0497	3.5255
RSM_22	34.7	3.3	16.1	23.7	3.0601	3.5249
RSM_23	29.9	2.9	14.2	19.6	3.0509	3.5184
RSM_24	36.2	2.3	15.9	23.5	3.0609	3.5195
RSM_25	34.1	3.5	16.2	24.5	3.0604	3.5255
RSM_26	35.7	2.8	16.5	22.2	3.0573	3.5201

We used data from Table 2 to evaluate the *H*_*C2*_ response surface as a function of the synthesis parameters. The best-fit surface was assessed with the software STATISTICA from StatSoft neglecting third-order interactions between the synthesis parameters^31^, as per the following quadratic polynomial:

1 $$\mu_{0} H_{C2} \left( {T_{d} ,HR,t,P} \right) = {\text{A}}_{0} + {\text{A}}_{1} T_{d} + {\text{A}}_{2} T_{d}^{2} + {\text{A}}_{3} HR + {\text{A}}_{4} HR^{2} + {\text{A}}_{5} t + {\text{A}}_{6} t^{2} + {\text{A}}_{7} P + {\text{A}}_{8} P^{2} + {\text{A}}_{9} T_{d} HR + {\text{A}}_{10} T_{d} t + {\text{A}}_{11} T_{d} P + {\text{A}}_{12} HR t + {\text{A}}_{13} HR P + {\text{A}}_{14} t P.$$

The best-fit values of the coefficients “${A}_{i}$” were evaluated by the least squares method and are listed in Table 3. For the RSM-DoE, we kept the Ar-jet pressure constant to its maximum value.

**Table 3:** Best-fit values of the coefficients ${\mathrm{A}}_{\mathrm{i}}$ of the $\it {{H}}_{{C}2}$ response surface at 10 K, as per Eq. (1).

A_0_ =	− 9.978E+01 T	A_5_ =	3.251E−01 T min^−1^	A_10_ =	− 1.062E−04 T °C^−1^ min^−1^
A_1_ =	1.912E−01 T °C^−1^	A_6_ =	− 1.285E−03 T min^−1^	A_11_ =	− 1.388E−04 T °C^−1^ MPa^−1^
A_2_ =	− 6.767E−05 T °C^−2^	A_7_ =	1.063E−01 T MPa^−1^	A_12_ =	1.646E−05 T °C^−1^
A_3_ =	1.575E−02 T min °C^−1^	A_8_ =	1.141E−05 T MPa^−2^	A_13 _=	3.447E−05 T min °C^−1^ MPa^−1^
A_4_ =	− 3.269E−06 T min^2^ °C^−2^	A_9_ =	− 2.108E−05 T min °C^−2^	A_14_ =	− 1.770E−04 T min^−1^ MPa^−1^

**Figure 1:**
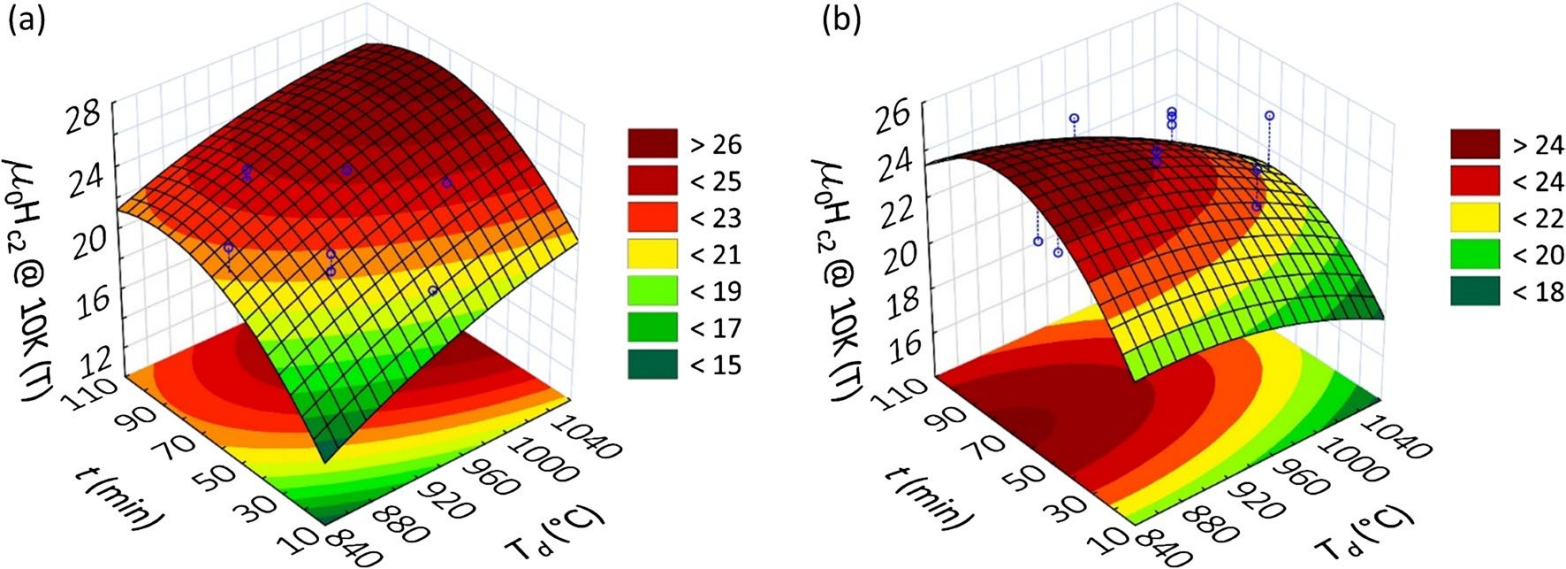
*H*_*C2*_(*T*_*d*_,*t*) response surface at 10 K in the synthesis-parameter ranges where the DoE predicts the presence of relative maxima. Panel (**a**) is a slice of the *H*_*C2*_(*T*_*d*_,*HR*,*t*,*P*) surface for fixed values of *HR* (700 °C/min, “0” in coded units) and *P* (125 MPa, “α−” in coded units). From this plot it is deduced that at low pressure *H*_*C2*_ is maximized for *T*_*d*_ $\gtrsim$ 950 °C and 60 min $\lesssim$ *t* $\lesssim$ 90 min. Panel (**b**) is a slice of *H*_*C2*_(*T*_*d*_,*HR*,*t*,*P*) for fixed values of *HR* (1000 °C/min, “+” in coded units) and *P* (375 MPa, “α+” in coded units). From this plot it is deduced that at high pressure *H*_*C2*_ is maximized for *T*_*d*_ $\lesssim$ 950 °C and 60 min $\lesssim$ *t* $\lesssim$ 90 min.

Equation (1) allowed us to identify the input-parameter combinations that maximize *H*_*C2*_ at 10 K. *H*_*C2*_(*T*_*d*_,*HR*,*t*,*P*) presents maxima in two synthesis-parameter regions characterized by: (1) high reaction temperature and low pressure (*T*_*d*_ > 900 °C and *P* < 250 MPa), (2) low reaction temperature and high pressure (*T*_*d*_ < 900 °C and *P* > 250 MPa). In order to allow the visualization the two maxima, panels (a) and (b) of Fig. 1 report two slices of the *H*_*C2*_(*T*_*d*_,*HR*,*t*,*P*) response surface performed at *HR* = 700 °C/min and *P* = 125 MPa, *HR* = 1000 °C/min and *P* = 375 MPa, respectively. The highest maximum (*µ*_*0*_*H*_*C2*_ ~ 26 T) is expected when combining high *T*_*d*_ and low *P*. Based on the predictions of the RSM DoE, we prepared further 7 samples selecting synthesis conditions favorable for *H*_*C2*_. Details about the preparation conditions for this “post-DoE” batch are reported in Table 10 of Methods. Superconducting and lattice parameters are reported in Table 4.

**Table 4:** Superconductivity and lattice parameters of the “post-RSM-DoE” sample batch. Estimated standard deviation for *a* and *c* is $\lesssim$ 0.0001 Å for all samples.

Sample ID	*T*_*c*_ (K)	*ΔT*_*c*_ (K)	*H*_*irr*_* @*10 K (T)	*H*_*C2*_* @*10 K (T)	*a* (Å)	*c* (Å)
M_1	35.2	2.2	20.0	24.9	3.0579	3.5232
M_2	33.7	5.7	11.8	23.2	3.0610	3.5266
M_3	35.9	1.7	19.1	23.7	3.0754	3.5258
M_4	37.3	0.6	16.2	20.2	3.0813	3.5257
M_5	36.9	0.9	16.4	21.8	3.0777	3.5265
M_6	37.2	0.6	14.4	19.2	3.0807	3.5268
M_7	37.4	0.7	16.7	22.1	3.0811	3.5297

The manufacturing process adopted in this work includes the flattening by uniaxial pressing of the SS tube filled with the precursors. It has been shown that non-hydrostatic cold-deformation processes may lead to a partial texturing of the MgB_2_ crystallite *c*-axis along the applied-pressure direction^32,33^. All samples from this study were investigated with the magnetic field perpendicular (⊥) to the uniaxial-pressure direction. Selected samples with the best in-field performances were further investigated for magnetic fields parallel (//) to the pressure direction. This short list includes samples from the RSM DoE (samples RSM_1, RSM_17, RSM_25) and from the post-RSM-DoE batch (samples M_1 and M_3). Obtained *H*_*C2*_ and *H*_*Irr*_, as evaluated at 10 K for the two orientations of the field, are reported in Table 5. In polycrystalline samples, *R(H)* measurements allow one to probe *H*_*C2*_ for fields perpendicular to the crystallographic *c* axis, ${H}_{C2}^{\perp c}$, regardless of the applied-field direction. Since ${H}_{C2}^{\perp c}$>$H_{C2}^{//c}$, upon decreasing the applied field *R(H)* starts devting from its normal state value as soon as $H \le H_{C2}^{ \bot c}$, because grains with the *c*-axis perpendicular to the external-field direction become superconducting and do not contribute to the electrical resistance anymore. The variability between *H*_*C2*_ values of Table 5 measured for the two field orientations is below ~ 5% and has to be considered as an experimental uncertainty inherent to the procedure adopted to determine *H*_*C2*_. *H*_*Irr*_ is expected to be independent of the magnetic-field orientation in untextured samples, whilst a dependence is expected in textured or weakly-textured specimens^34^. In particular, higher *H*_*Irr*_ should be measured when *H* is perpendicular to the uniaxial-pressure direction^34^. In the case of the sample M_1, we measured *H*_*Irr*_ higher by ~ 15% in this orientation. This difference is above the measurement uncertainty of ~ 5% and is an indication of weak *c*-axis texturing along the applied-pressure direction^34^.

**Table 5:** *H*_*Irr*_ and *H*_*C2*_ values as measured in a selection of samples for two different orientations of the magnetic field, parallel and perpendicular to the uniaxial pressure direction.

Sample ID	*µ*_*0*_*H*_*irr*_ (10 K) ⊥ (T)	*µ*_*0*_*H*_*irr*_ (10 K) // (T)	*µ*_*0*_*H*_*C2*_ (10 K) ⊥ (T)	*µ*_*0*_*H*_*C2*_ (10 K) // (T)
RSM_1	18.0	18.8	23.6	23.0
RSM_17	18.1	17.0	24.0	24.9
RSM_25	16.2	16.7	24.5	23.4
M_1	20.0	17.1	24.9	26.2
M_3	19.1	17.5	23.7	23.3

## Discussion

### Record high *H*_*C2*_ and *H*_*Irr*_

**Figure 2:**
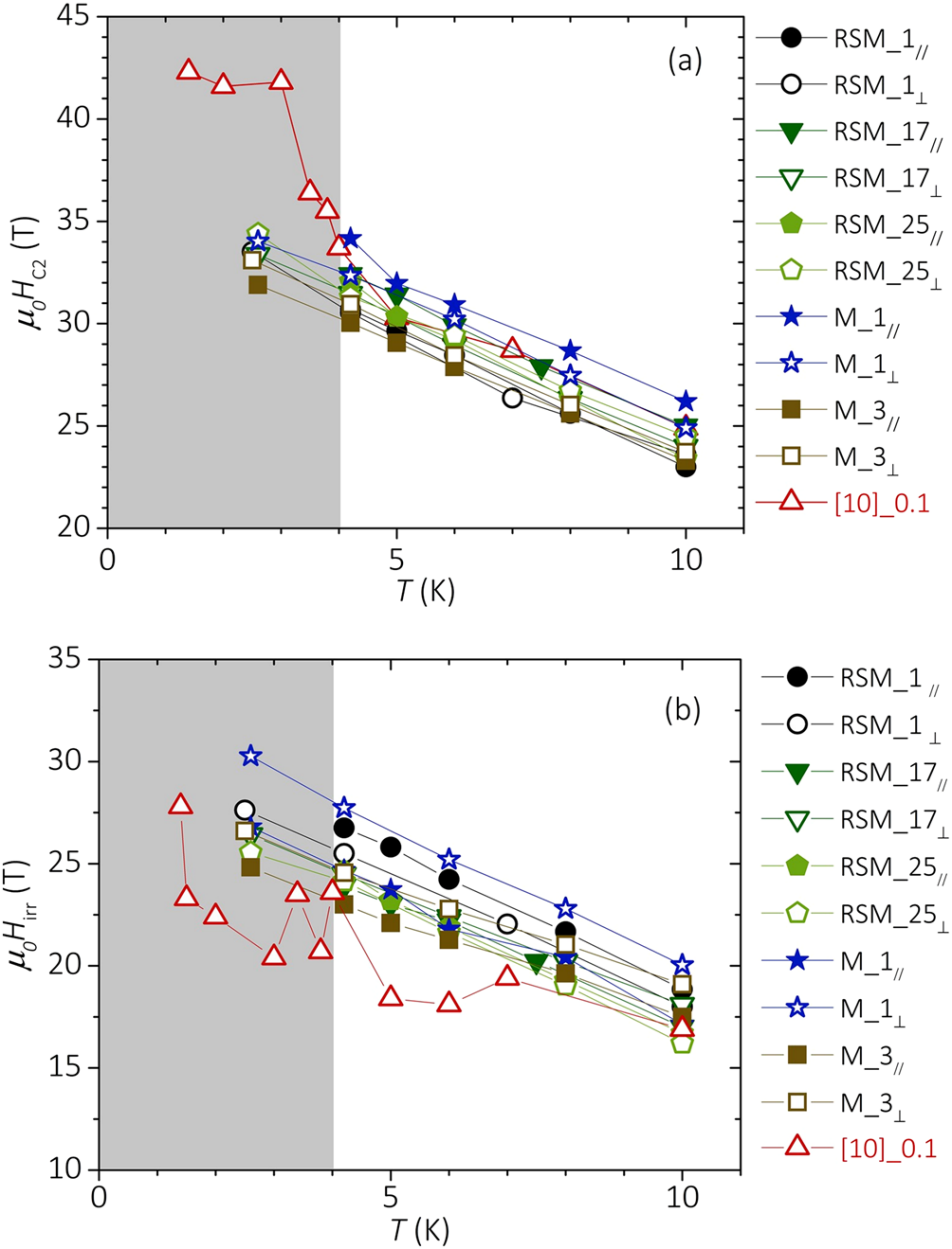
Temperature dependence of the upper critical field (**a**) and of the irreversibility field (**b**) for the best-performing samples from this work. Data of the record-high *H*_*C2*_ bulk sample from^10^ are reported for comparison.

Figure 2 presents the temperature (*T*) dependence of *H*_*C2*_ (panel (a)) and *H*_*Irr*_ (panel (b)) for the best-performing samples from this work listed in Table 5. For comparison, we included in the plot the curves corresponding to the polycrystalline sample with the highest *H*_*C2*_ from [10], which was prepared with a nominal C content x = 0.1 (hereafter, we refer to this sample as “[10]_0.1”). No texturing is expected for [10]_0.1 as it was reacted in the absence of any external pressure. We highlighted in the graph two zones associated with temperatures higher (white background) or lower (grey background) than 4 K. At *T* ≥ 4 K, the rapid-synthesis route allowed us to achieve *H*_*C2*_ values comparable with the record-high *H*_*C2*_ of [10]_0.1, for various combinations of the synthesis parameters. This is good news because it indicates that the synthesis conditions leading to *μ*_*0*_*H*_*C2*_ above 30 T at 4.2 K and above 23 T at 10 K can be adapted to consider specific manufacturing requirements. Furthermore, data from [10] represent the result of a single experiment that was never replicated. At *T* ≥ 4 K, *H*_*C2*_ measured in the sample M_1 in parallel field overcomes that of the sample [10]_0.1 and thus set a new record for *H*_*C2*_ in MgB_2_ bulk samples. At *T* < 4 K, the *H*_*C2*_(*T*) curves of samples from this work stay all below that of [10]_0.1. Furthermore, we do not observe any sudden increase of *H*_*C2*_ at *T* $\lesssim$ 2 K. The temperature dependence of *H*_*Irr*_ is reported in Fig. 2b. Samples reacted by the rapid-synthesis route exhibit higher *H*_*Irr*_ with respect to [10]_0.1. The difference is up to ~ 8 T in the case of the sample M_1. This result is important for applications since *H*_*Irr*_ defines the operational limits in superconducting magnets. *H*_*Irr*_ seems to extrapolate linearly down to *T* = 0 K. In the case of untextured MgB_2_ bulk samples prepared by standard synthesis routes, it is typically observed *H*_*Irr*_ ~ 0.5 *H*_*C2*_^35,36^. Data of Tables 1, 2 and 4 allow one to deduce that ~ 70% of the samples from this work have *H*_*Irr*_ $\gtrsim$ 0.7 *H*_*C2*_. In the framework of the anisotropic Ginzburg–Landau theory, *H*_*Irr*_ of untextured samples is described by:

2 $$H_{Irr} = \frac{{H_{C2}^{ \bot c} }}{{\sqrt {\left( {\gamma^{2} - 1} \right)p_{c}^{2} + 1} }},$$

where *γ*=${H}_{C2}^{\perp c}/{H}_{C2}^{//c}$ is the upper-critical field anisotropy, and *p*_*c*_ is the percolation threshold, which represents the minimum superconducting-grain fraction for a continuous path through the superconductor^34,37^. In granular superconductors, *p*_*c*_ depends on the coordination number, i.e., on the average number of grain first neighbors (that in turn depends on the grain packing density), but also on the presence of insulating spurious phases. *p*_*c*_ can thus be considered as an indicator of the electrical connectivity, *K*, in a superconductor: the higher *p*_*c*_, the lower *K*. Data of Table 5 show that the difference in *H*_*Irr*_ measured for the two orientations of *H* is clearly above the experimental uncertainty only in the case of the sample M_1. For the other samples listed in Table 5, we can in a first approximation assume that texturing effects are negligible and make use of Eq. (2) to evaluate *p*_*c*_. The low-temperature value of *γ* can be estimated from the sample critical temperature by the following empirical expression valid also for C-doped samples, which was derived in^18,38^ comparing results obtained in a large set of samples with different *T*_*C*_:

3 $$\gamma = \frac{{t_{c}^{2} + 16.7t_{c} \left( {1 - t_{c} } \right)}}{{3.88 - 3.724t_{c} }}.$$

**Figure 3:**
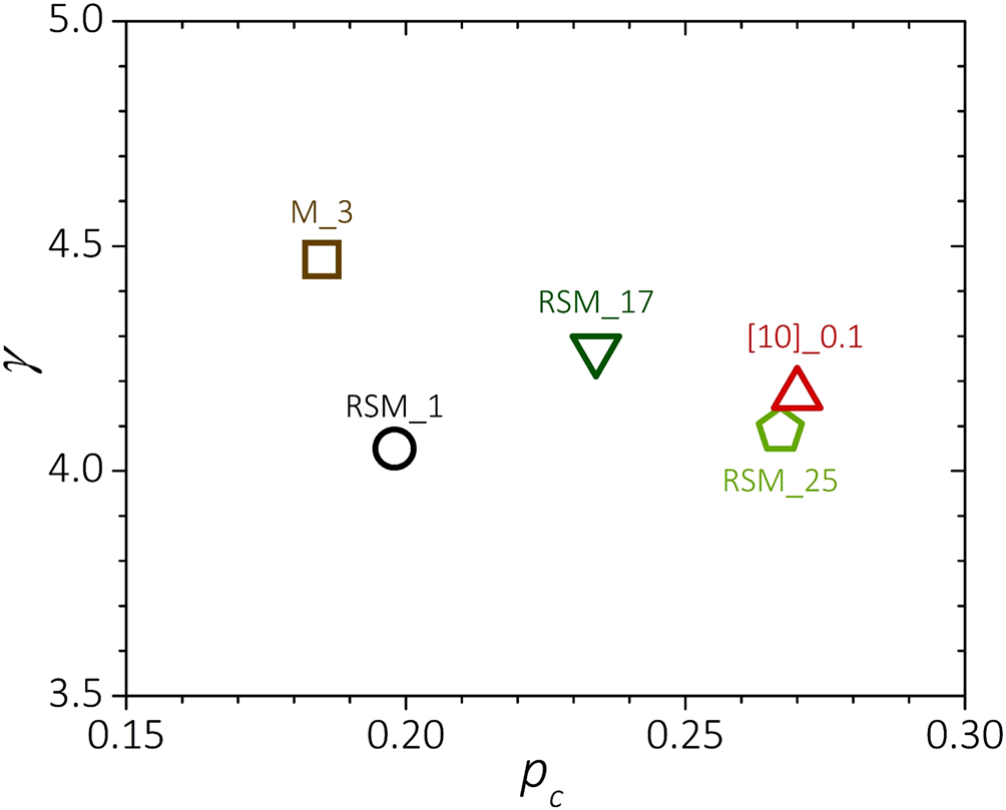
Low-temperature upper-critical-field anisotropy (*γ*) and percolation threshold *p*_*C*_ calculated as described in the text for the samples RSM_1, RSM_17, RSM_25, M_3 from this work and the record-high-*H*_*C2*_ sample from^10^.

Here ${t}_{c}$ = ${T}_{c}$/${T}_{c0}$ and ${T}_{c0}$ = 39.43 K is the *T*_*C*_ expectation for samples in the clean limit^18,38^. Having estimated $\gamma$, ${p}_{c}$ can be calculated by Eq. (2) using for ${H}_{C2}^{\perp c}$ and *H*_*Irr*_ the experimental results reported in Table 5, averaging the values obtained in the two field orientations. Figure 3 reports ${p}_{c}$ and $\gamma$ evaluated at 10 K using Eqs. (2) and (3). Samples prepared by the rapid-synthesis route have ${p}_{c}$ smaller than that of sample [10]_0.1. Reported values are more generally low even when compared with further results from the literature for ${p}_{c}$, which is typically $\gtrsim$ 0.25 for C-doped samples^37–40^. This result indicates that the higher *H*_*Irr*_ measured in the samples prepared with the rapid synthesis route has to be ascribed to a better electrical connectivity between the superconducting grains. This conclusion is further supported by the high values measured for the sample mass density. About 90% of the samples prepared in this work have mass density $\gtrsim$ 2.1 g/cm^3^, which is ~ 80% of the theoretical value for MgB_2_^41^.

### Effects of C doping on the crystal structure and electronic properties

**Figure 4:**
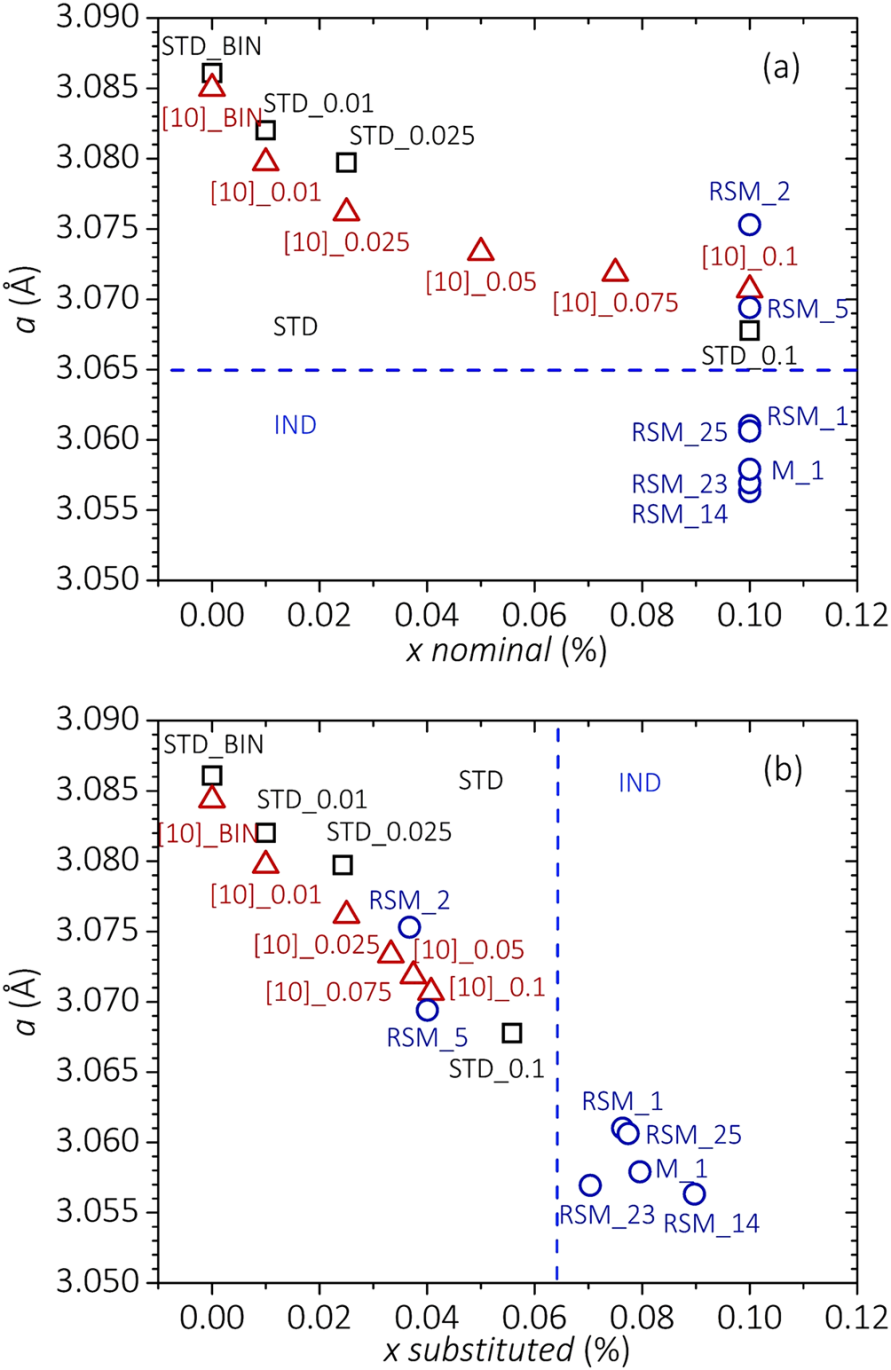
Dependence of the a lattice parameter on the nominal C content (**a**) and with the effective substituted C content (**b**). The two panels include data from this work and from^10^. Lattice parameter uncertainty is smaller than the symbols’ size.

We evaluated by X-ray powder diffraction experiments the *a* and *c* lattice parameters for all the samples produced in this campaign. Values are reported in Tables 1, 2 and 4. On the atomic scale, C can be substituted for B in the MgB_2_ crystal structure, or remain interstitial within B rings^22,23^. Using the MgB_2_ phase as a reference state, the enthalpy of formation at 0 K is negative in the case of C substitution, positive for interstitial C^23^. Therefore, substitutional insertion is energetically favored. C atoms can also be segregated outside the superconducting grains, thus impacting extrinsic superconducting properties such as grain connectivity or vortex pinning^21,42^. Experimental and theoretical works have shown that lattice parameter *a* decreases upon augmenting x in bulk Mg(B_1−x_C_x_)_2_ samples, whilst *c* remains nearly constant^22,23^. In Mg(B_1−x_C_x_)_2_ films, both *a* and *c* are observed to increase with the C-doping content^22,23^. Figure 4a reports the experimental dependence of *a* on the nominal amount of C doping. We included in this chart a binary sample (BIN-STD_1) and three IMD bulk samples reacted in our laboratory using a conventional muffle furnace (STD_0.01, STD_0.025, STD_0.1), binary and DWCNT-doped samples from [10] ([10]_ BIN, [10]_0.01, [10]_0.025, [10]_0.05, [10]_0.1), and a selection of samples from the RSM DoE, that are representative of the *a* variability in our experiment. Samples reacted with conventional furnaces both from this work and from the literature show that *a* decreases upon increasing the nominal amount of C. However, *a* saturates at values approaching ~ 3.065 Å for x ~ 0.1, as also reported in^42–45^. The rapid-synthesis route leads to a large variability of *a* in spite of the same nominal doping (x = 0.1). Very interestingly, most of the samples prepared with this process have *a* < 3.065 Å, indicating that the rapid-synthesis route allows for the substitution of a larger fraction of C into the B sites, at a same nominal doping. As a general trend, we found that low *a* values are typically associated with high *T*_*d*_. The fact that high dwell temperatures are beneficial for C substitution in MgB_2_ agrees with further results from the literature^44,46^. A dedicated study would be needed to draw definitive conclusions about the microscopic mechanisms that lead to a more efficient C substitution when using the rapid-synthesis route. On the other hand, we can infer that the rapid heating and cooling (quench), which are unique characteristics of the employed route, play a certain role in enhancing the C-substitution efficiency with respect to conventional synthesis methods. In particular, it is possible that the C segregation out of the grains during a slow cooldown is hindered by the post-reaction quench.

**Figure 5:**
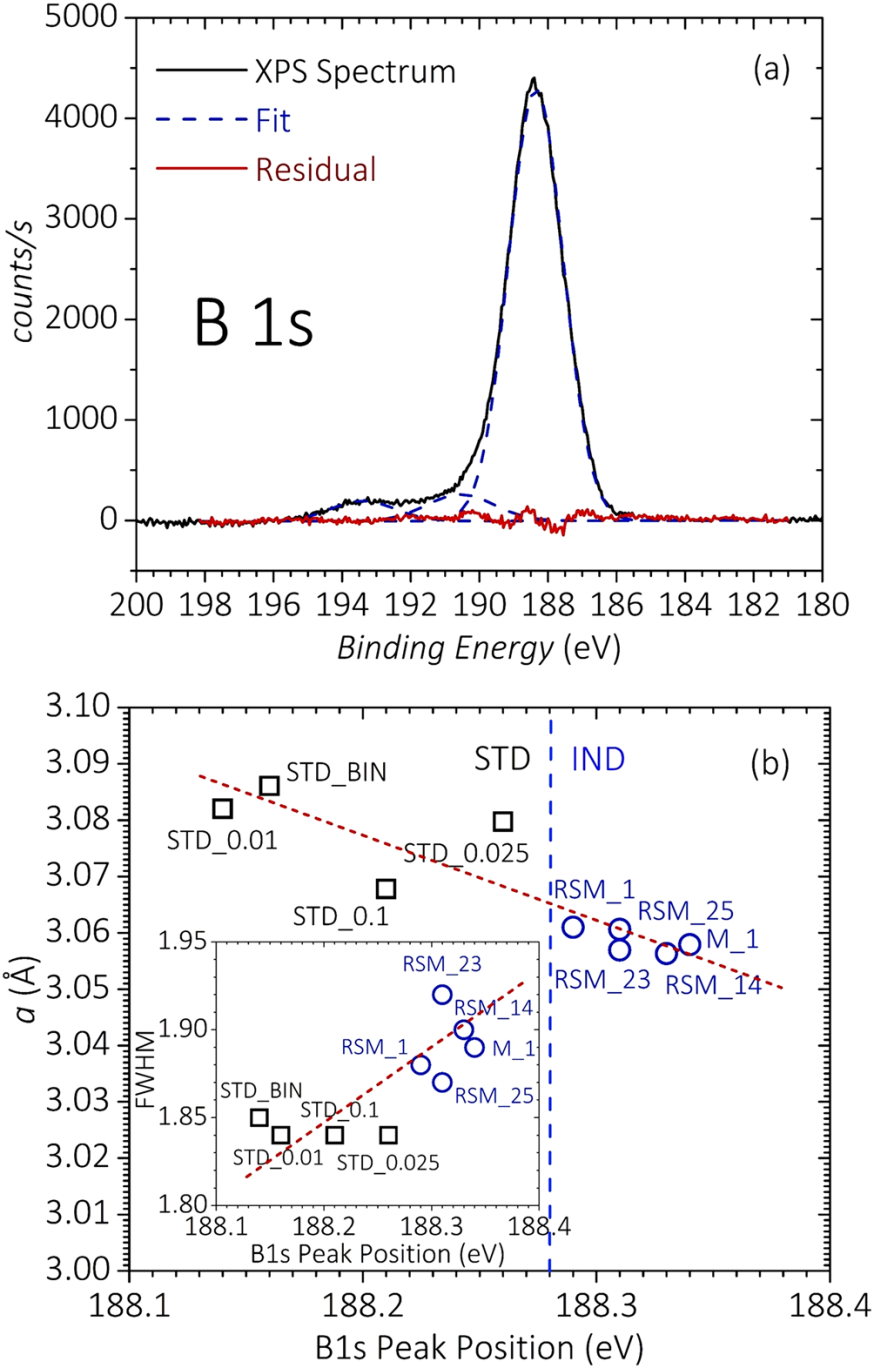
(**a**) XPS B 1s spectrum of sample M_1 along with the Gaussian-peak best-fit curves. A linear background has been subtracted from the raw spectrum before fitting. (**b**) Variation of the lattice parameter *a* with the main-peak position of the B 1s spectrum. The inset shows the correlation between the main-peak position and its FWHM. Dashed straight lines are eye-guide lines.

Comparison between neutron-diffraction experiments and X-ray analyses has previously demonstrated that the actual level of C substitution in Mg(B_1–x_C_x_)_2_ can be estimated as x≈7.5⋅Δ(*c*/*a*), where Δ(*c*/*a*) is the change in *c*/*a* compared to a pure sample^21,44,47^. The variation of *a* as a function of the actual substituted-C content is reported in Fig. 4b. Effective C substitution up to 90% of the nominal DWCNT content is reached with the rapid-synthesis route. The effectiveness of this route in substituting C for B is further confirmed by XPS, which we carried out on a batch of five samples prepared by the rapid-synthesis route (RSM_1, RSM_14, RSM_23, RSM_25, M_1) and four samples prepared using a standard furnace (STD_BIN, STD_0.01, STD_0.025, STD_0.1). Figure 5a shows the B 1s spectrum for the sample M_1, together with the result of least squares fitting of the spectrum considering pure Gaussian-line shapes, in agreement with previous reports^48,49^. The B 1s spectrum is composed of three peaks centered at ~ 188.3 eV, ~ 190.5 eV and ~ 193.5 eV. In agreement with other XPS reports, we assign the main peak located at 188.3 eV to B in MgB_2_, and the two peaks at 193.5 eV and 190.5 eV to B_2_O_3_ and other contaminants of B, respectively^11,48,50,51^. The B 1s spectra of all measured samples are qualitatively similar and show only a single broad peak associated with MgB_2_ or Mg(B_1−x_C_x_)_2_. Interestingly, as shown in Fig. 5b, the binding energy of this peak increases as the *a* lattice constant contracts. Samples reacted with the rapid-synthesis route which have *a* < 3.065 Å exhibit a B 1s peak position located at up to ~ 0.2 eV higher binding energy compared to the samples with the larger *a* values. This binding-energy change can be attributed to the shift of the Fermi level due to the additional electrons doped into the system when substituting C for B^20,47^. Therefore, Fig. 5b provides further evidence that substitutional C doping is higher for the samples prepared by the rapid-synthesis route. Since, as shown in Fig. 4, effective substitution in our samples is always less than 0.1, the maximum Fermi-level shift of ~ 0.2 eV is consistent with the value of ~ 0.3 eV theoretically predicted for effective x = 0.1^52^. The inset of Fig. 5b reports the best-fit values of the full width at half maximum (FWHM) as a function of the peak position for the B 1s peak attributed to MgB_2_. The FWHM increases with the binding energy, which could be due to an increasing contribution from a peak component associated with B-C bonding, further confirming the increased substitution of C in the MgB_2_ lattice.

### Correlation between *H*_*C2*_ and *T*_*C*_ with the *a* lattice parameter

**Figure 6:**
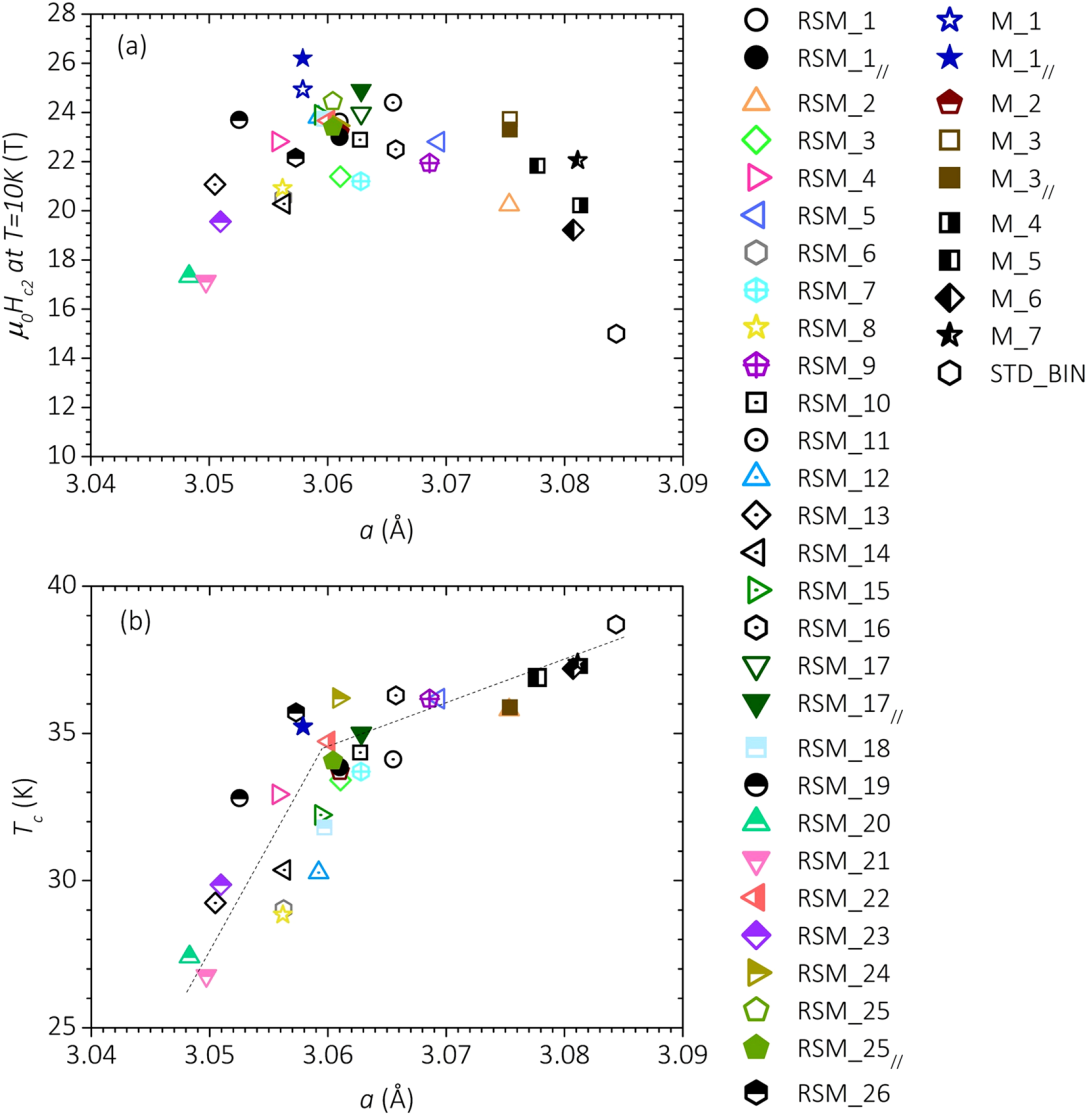
Correlation between *H*_*C2*_ at 10 K (**a**) and *T*_*C*_ (**b**) with the lattice parameter a. The highest *H*_*C2*_ values are achieved when a is ~ 3.06 Å. For the same a value, a marked change in the slope of the *T*_*C*_ vs a dependence is observed. Dashed straight lines are eye-guide lines.

Substituted, interstitial or intergranular C can affect differently the intraband and interband scattering rates. C substitution should primarily lead to an increase of the σ-band intraband scattering, whilst grain boundaries should affect the scattering rates on both σ and π bands^24^. No study reports on the role of the energetically unfavorable interstitial C on the scattering rates. The lattice parameter *a* can be used as a sort of caliper to measure the C substitution in the MgB_2_ lattice. Figure 6a shows the correlation between *H*_*C2*_ at 10 K and *a* for all samples investigated in this study. Starting from the binary sample located at the bottom-right corner of the chart, one observes that *H*_*C2*_ initially increases upon lowering *a*, it reaches a maximum when *a* ~ 3.06 Å and finally decreases for *a* $\lesssim$ 3.06 Å. The enhancement of *H*_*C2*_ upon increasing the effective C doping has to be mainly ascribed to an increased intraband scattering, as further documented in the literature^15,16,20^. The introduction of C atoms in the MgB_2_ structure also leads to a reduction of *T*_*C*_, which is steeper for *a* $\lesssim$ 3.06 Å as shown in Fig. 6b. Band filling due to electron doping is expected to lower *T*_*C*_^53^. In particular, a linear decrease of *T*_*C*_ with *x* in Mg(B_1−x_C_x_)_2_ is theoretically predicted for doping levels up to *x* ~ 0.15, if changes in the bands and phonon spectrum due to the elemental doping are considered^53^. Our experimental observation that the slope of the *T*_*C*_ vs *a* dependence changes for *a* $\lesssim$ 3.06 Å, which corresponds to *x* ~ 0.08, suggests that the effects of interband scattering cannot be ruled out in our series of samples, at least for those samples with *a* $\lesssim$ 3.06 Å. The enhancement of the interband scattering rate because of substitution of C for B has been further documented in the literature^15,53^. In spite of the loss in condensation energy due to the lower *T*_*C*_, moderate levels of lattice deformation characterized by *a* in the range ~ 3.06 Å to  ~ 3.08 Å lead to a net gain in terms of *H*_*C2*_. A further decrease of the lattice parameter *a* (*a* $\lesssim$ 3.06 Å) results in a reduction of *H*_*C2*_. Analogous conclusions are drawn when analyzing the evolution of *H*_*C2*_ at 4.2 K with *a*.

**Figure 7:**
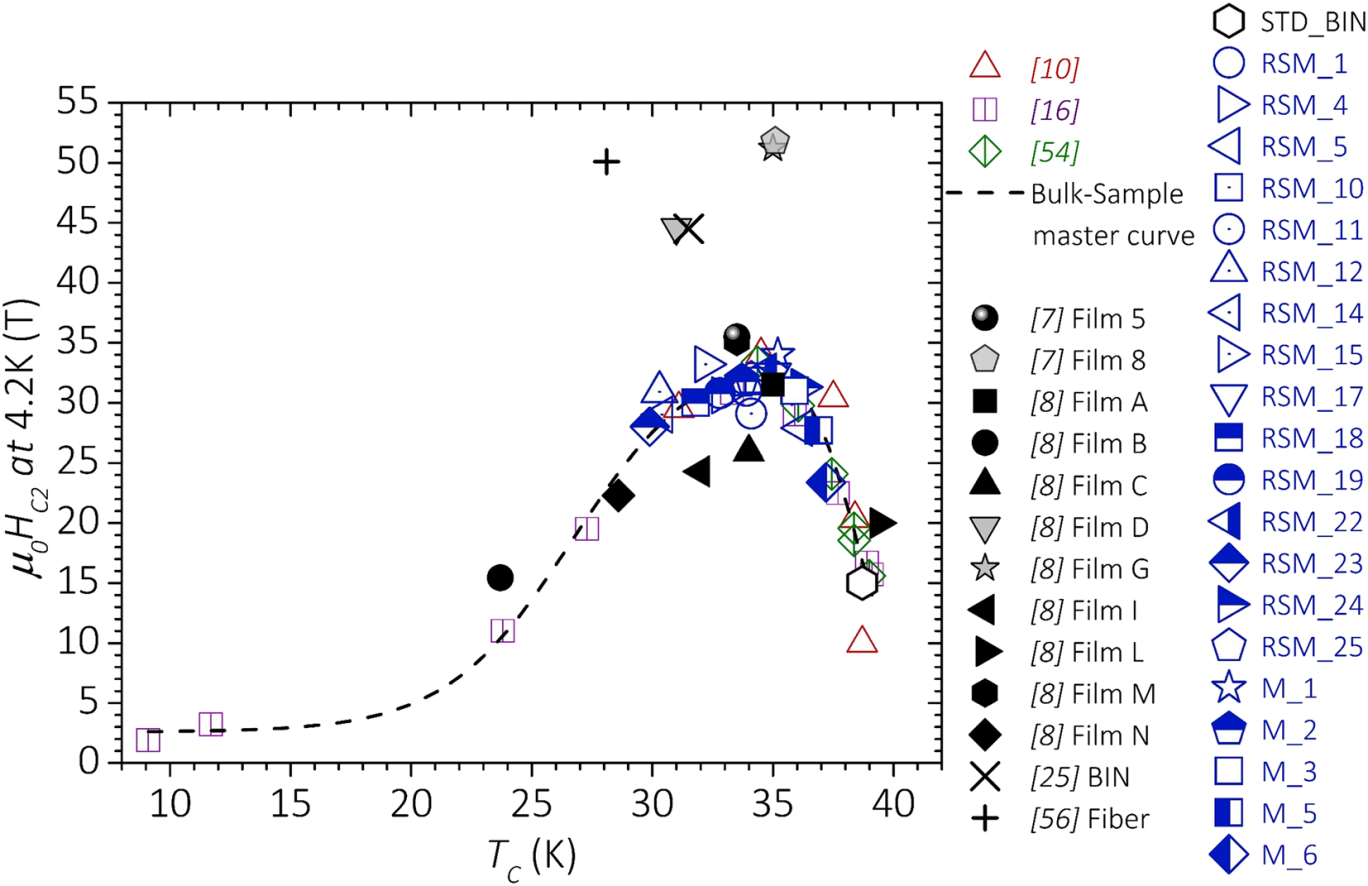
Correlation between *H*_*C2*_ (4.2 K) and *T*_*C*_ for bulk samples from this work and from the literature. The dashed line represents the best fit curve of all experimental data obtained in bulk samples, as described in the text.

Figure 7 reports the correlation between *H*_*C2*_(4.2 K) and *T*_*C*_. We included in the chart all samples from this work investigated at *T* = 4.2 K and data available in the literature for C-doped^10,54^ and irradiated bulk samples^16,55^. A binary bulk prepared in our laboratory (STD_BIN) was added as a reference. In order to allow a comparison with the results obtained in films, we also included data of C-doped films^7,8^, of a high-disorder binary film^25^, and of a 0.75 μm-thick polycrystalline coated conductor deposited on SiC fibers that all showed *µ*_*0*_*H*_*C2*_ ~ 50 T^56^. In the case of the films, we reported only *H*_*C2*_ data measured with the field parallel to the surface (the highest values). Indeed, *R*(*H*) experiments carried out on polycrystalline samples provide an estimation of ${H}_{C2}^{\perp c}$^34^. In bulk samples, *H*_*C2*_ is maximized when *T*_*C*_ is in the range 34 K ± 2 K, regardless of the specific source of disorder (C doping, irradiation, synthesis conditions). All experimental *H*_*C2*_(4.2 K) data of bulk samples can be predicted from *T*_*C*_ with an uncertainty below ~  ± 20% by an asymmetric 2-sigma function (dashed curve in Fig. 7):

4 $$\upmu _{0} H_{C2} \left( {{4}.{2} {\text{K}}} \right) = {\text{A}} + {\text{B}}*\left( {{1}/\left( {{1} + {\exp}\left( { - \left( {T_{C} - {\text{T}}_{0} + {\text{w}}_{{1}} /{2}} \right)/{\text{w}}_{{2}} } \right)} \right)} \right)*\left( {{1} - {1}/\left( {{1} + {\exp}\left( { - \left( {T_{C} - T_{0} - {\text{w}}_{{1}} /{2}} \right)/{\text{w}}_{{3}} } \right)} \right)} \right).$$

The parameters’ best-fit values and their statistical errors, as determined by least squares fitting, are A = 2.6 T ± 1.5 T, B = 31.2 T ± 2.4 T, T_0_ = 32.5 K ± 0.3 K, w_1_ = 12.0 K ± 0.7 K, w_2_ = 2.4 K ± 0.5 K, w_3_ = 1.1 K ± 0.2 K. The dashed curve of Fig. 7 can be considered as an empiric master curve for the *H*_*C2*_ vs *T*_*C*_ dependence in MgB_2_ bulk samples in which disorder has been induced by doping, irradiation or synthesis conditions. At odds with what observed in bulk MgB_2_, a large variability of *H*_*C2*_ is found for films with similar *T*_*C*_. No correlation between *H*_*C2*_ and the lattice constants *a* or *c* is reported for C-doped films^7,8^. What is thus the origin of the very-high upper critical field (*μ*_*0*_*H*_*C2*_(4.2 K) > 40 T) measured in MgB_2_ films? We found that only three samples out of all the C-doped films reported in the literature have measured *H*_*C2*_(4 K) values distinctly above the bulk-sample master curve^7,8^. C doping cannot be considered the only key to achieve high *H*_*C2*_ because the binary film from^25^ prepared by pulsed-laser deposition showed *μ*_*0*_*H*_*C2*_(4.2 K) ~ 44.5 T. Therefore, even if this and other studies prove that C doping can significantly enhance *H*_*C2*_ with respect to binary samples, there has to be a specific “type of disorder” able to unlock much higher *H*_*C2*_ values. The two-band theory suggests that *H*_*C2*_ can be significantly enhanced at low temperatures if the π band is dirtier than the σ band^15^. In this case, an upward curvature of *H*_*C2*_ vs *T* is expected. Contrary to what reported in^10^, our results do not give evidence of an upward curvature of *H*_*C2*_(*T*) at low *T*. This indicates that C substitution in Mg(B_1−x_C_x_)_2_ does not selectively increase the π-band intraband scattering rate, in agreement with theoretical expectations^24^. In view of the lower amount of substituted C in the samples from^10^ with respect to those from this work, one could argue that interstitial C is at the origin of the observed upward curvature. However, the presence of interstitial C was not proven in^10^ and C should be more probably segregated outside the superconducting grains^45^. We found that the films of Fig. 7 that have *H*_*C2*_ above the bulk-sample master curve share a fiber texture, which is characterized by a rotational degree of freedom of the crystallographic *a* axis around the *c* axis^57^. No information about the type of texture is reported for the film 8 from^7^, which has *μ*_*0*_*H*_*C2*_(4.2 K) ~ 52 T. Zhu et al*.* observed in their film a tilt at the nanometric scale of the *c* axis induced by C doping and pointed it out as the possible cause of the very-high *H*_*C2*_^24^. This kind of disorder may likely perturb the B p_z_ orbitals from which the π band arises^12,24^, and possibly enhances selectively the intraband scattering in this band. It is possible that the same kind of lattice distortion is achieved in those of the C-doped films that present a fiber texture along the *c* axis. *c*-axis disorder due to nanometric inclusions was also reported for the binary MgB_2_ film showing record-high *H*_*C2*_^25,58^. Further studies about the correlation between microstructural properties of films and *H*_*C2*_ should be carried out to achieve a more complete understanding of the mechanisms responsible of the record *H*_*C2*_ values. This study points out that in bulk samples the introduction of disorder by different sources enhances both the intraband and interband scattering rates leading to an upper limit for *μ*_*0*_*H*_*C2*_(4.2 K) of ~ 35 T. In the case of the thin films and of the coated conductor deposited on SiC fibers, it is another type of structural defect that leads to *μ*_*0*_*H*_*C2*_(4.2 K) ~ 50 T, while keeping *T*_*C*_ above 25 K.

## Conclusions

We presented the results of a wide experimental campaign to investigate the role of carbon doping in the enhancement of *H*_*C2*_ in MgB_2_ bulk samples. The main purpose of this work was understanding whether the very-high *μ*_*0*_*H*_*C2*_ values of ~ 50 T at 4.2 K, as observed in disordered films, can also be achieved in polycrystalline samples. This would allow widening the application domain of MgB_2_ in magnet technology. Based on theoretical and experimental evidences that structural disorder is the key to enhance *H*_*C2*_, we produced samples by a rapid-synthesis route, which allowed us to explore ranges of variation of the synthesis conditions not achievable with traditional techniques. In particular, we quenched the synthesis process with the idea of freezing the system in out-of-equilibrium configurations. The study was guided by a Design of Experiment. This statistical tool allowed us to characterize the *H*_*C2*_ surface response as a function of the synthesis parameters. We defined different regions of the synthesis-parameter space that maximize *H*_*C2*_. Experimental *H*_*C2*_ data reflected with good precision and reproducibility the predictions of the DoE. X-ray and XPS analyses demonstrated that the rapid-synthesis route allows us to reach levels of C substitution in the B sites not achievable with conventional manufacturing routes for bulk samples. Furthermore, we documented record-high *H*_*Irr*_ resulting from a good electrical connectivity between superconducting grains. This is an important result of this work, since *H*_*Irr*_ represents the maximum field at which a superconductor can be operated in superconducting magnets. In spite of the enhanced degree of C substitution, *µ*_*0*_*H*_*C2*_ appears to be bounded to maximum values of ~ 26 T and ~ 35 T at 10 K and 4.2 K, respectively. *T*_*C*_ and *H*_*C2*_ correlate well with the contraction of the *a* lattice parameter. *T*_*C*_ decreases monotonously upon increasing the structural disorder but its variation becomes much steeper for *a* $\lesssim$ 3.06 Å. This value of the lattice parameter *a* corresponds to the maximum of *H*_*C2*_, too. We also analyzed results reported in the literature for MgB_2_ in the form of films and coated conductors. Contrary to the case of bulk samples, it is not possible to define a master curve that allows estimating *H*_*C2*_ from *T*_*C*_. The two-band theory for *H*_*C2*_ demands for selective high scattering in the π band in order to achieve *µ*_*0*_*H*_*C2*_(4.2 K) as high as ~ 50 T. Our results indicate that C doping creates defects that act both as intraband and interband scattering centers, which respectively affect *H*_*C2*_ and *T*_*C*_. Furthermore, we did not observe any clear evidence of an upward curvature of *H*_*C2*_ at low *T*, as expected in the case of a π band much dirtier than the σ band. The type of disorder present in the films that showed very-high *H*_*C2*_ cannot be the same realized by C doping in bulk samples. Further investigations are needed to achieve a comprehensive understanding of this matter. Lattice deformations that produce a tilt of the *c* axis, which selectively affect the B p_z_ orbitals from which the π band arises, may be the key to achieve record-high *µ*_*0*_*H*_*C2*_ in the 50 T range at 4.2 K.

## Methods

### Sample preparation

In-situ MgB_2_ bulk samples were prepared using amorphous 99+% purity B powders, 99.9+% purity Mg turnings and 90+% purity DWCNT as precursors. We added 50 wt.% Mg excess to the reagents with respect to the stoichiometric ratio Mg:B = 1:2, as we previously proved that this is beneficial to the electrical connectivity of the samples^41^. Precursors were handled in glove box under inert atmosphere (pure Ar) to prevent oxygen contaminations. B powders (mixed with the DWCNT in the case of doped samples) were sandwiched between two Mg pellets inside an AISI-316-L stainless (SS) tube. The tube ends were closed by hydraulic press and sealed by Tungsten-Inert-Gas welding. The central part of the tube was subsequently submitted to uniaxial pressure in order to further densify the precursors before the reaction. Samples were reacted using a laboratory-made induction furnace described in^29^. The SS tube is inserted at the center of a water-cooled Cu coil, which is located inside a sealed chamber filled with Ar. The Cu coil induces currents in the SS crucible that acts as a susceptor. This allows reacting the precursors within the SS sheath with ramp rates as high as ~ 1000 °C/min. After the reaction dwell time, the synthesis process can be quenched by injecting Ar-gas at high pressure (up to 1.5 MPa). The sample temperature was recorded during the reaction using a pyrometer calibrated in the range 500–1200 °C. After reaction, we removed the SS sheath and cut samples of the desired size and shape by spark erosion. Typical dimensions of the samples used for electrical transport and structural characterizations are ~ 5 × 2 × 1 mm^3^.

### Screening DoE

We selected a 2^ k−1^ fractional factorial design for the screening DoE^59^. 2 is the number of levels for each factor (“−” and “ + ” in coded units) and *k* the number of factors or input variables. At this first stage, we let only 4 of the 5 input parameters vary, namely: heating ramp rate (*HR*), dwell temperature (*T*_*d*_), dwell time (*t*), pressure of the Ar-gas quenching jet (*ArP*). 2^* k*−1^ provides the number of experiments to be performed, which is 8 in our case. To this set of experiments, we added a “center point” (“0” in coded units) that represents the center value of all factors’ ranges. We replicated this run three times, preparing 11 samples in total. Tables 6 and 7 report the range of variation of the synthesis parameters and the specific samples’ synthesis conditions, respectively. Experiments were run in randomized order to guard against systematic biases.

**Table 6:** Range of variation of the synthesis parameters for the Screening DoE.

Coded Unit	−	0	+
*T* [°C]	850	900	950
*HR* [°C/min]	50	525	1000
*t* [min]	15	37.5	60
Ar jet pressure [MPa]	0	0.75	1.5

**Table 7:** Synthesis conditions (in coded units) of all the samples prepared for the Screening DoE. Coded units refer to Table 6.

Sample ID	Randomized order	*T *(coded units)	*H *(coded units)	*t *(coded units)	*Q *(coded units)
SCR_1	0	0	0	0	0
SCR_2	8	+	+	+	+
SCR_3	7	−	+	−	+
SCR_4	3	−	−	+	+
SCR_5	1	−	−	−	−
SCR_6	0	0	0	0	0
SCR_7	2	+	−	+	−
SCR_8	4	+	−	−	+
SCR_9	5	−	+	+	−
SCR_10	6	+	+	−	−
SCR_11	0	0	0	0	0

Samples SCR_1, SCR_6 and SCR_11 are three replicas of the DoE “center point”. Replicating the center point in a DoE provides a measure of process stability and reproducibility^30^. We found variations by ~ 1% for *T*_*C*_, ~ 40% for *ΔT*_*c.*_, ~ 2% for *H*_*Irr*_ and ~ 4% for *H*_*C2*_. The large spread of data found for *ΔT*_*c*_ does not seem to play a role in the variability of *H*_*C2*_. The highest *H*_*C2*_ values were found in samples whose synthesis process was quenched by injecting Ar gas at the highest pressure (1.5 MPa). This outcome agrees with results found in binary MgB_2_ samples prepared with the same technique^29^. Precursors did not react to form bulk samples when combining the lowest reaction temperature (850 °C) with the shortest dwell time (15 min). Therefore, it was not possible to characterize samples SCR_3 and SCR_5.

### Response-surface-methodology DoE

We selected a 2^k^ full factorial design augmented with center points and axial points (denoted by α+ and α− in coded units). Axial points are outside the input-parameter hypercube defined by the “−” and “ + ” levels. They are fundamental to build a second-order polynomial for the determination of the response surface ^31,59^. We used the following four input variables: heating ramp rate (*HR*), dwell temperature (*T*_*d*_), dwell time (*t*), pressure applied to the precursors before synthesis (*P*). On the basis of the screening-DoE results, we fixed the Ar-jet pressure to 1.5 MPa. In view of the the low variability observed for *H*_*C2*_ in the screening DoE, we performed only two replicas of the DoE center point (samples RSM_1 and RSM_7). Therefore, we performed 26 runs in total, composed by the 16 corners from the full factorial block (2^4^), 2 replications of the center point and 8 (2k) axial points. We run the DoE in randomized order. The two center-point replicas provided comparable values for all the investigated parameters but *ΔT*_*C*_, which varies by ~ 3.2 K in the two samples. Tables 8 and 9 report the range of variation of the synthesis parameters for all samples prepared in the frame of this DoE. The RSM DoE allowed us to evaluate the response surface of *H*_*C2*_(10 K) as a function of the four input variables. Based on the surface response predictions, we prepared further 7 samples with the aim of maximizing *H*_*C2*_ and verifing the predictions of the DoE. Preparations conditions for samples belonging to this “post-RSM-DoE batch” are reported in Table 10. Samples M_1 and M_2 are localized in proximity of the high-*T*_*d*_ and low-*P* maximum of the synthesis-parameter space, samples M_3 and M_4 to the low-*T*_*d*_ and high-*P* one. The response surface extrapolates towards high *µ*_*0*_*H*_*C2*_ of ~ 25 T at pressures higher than the upper boundary of the explored range (*P* > 375 MPa) for *T*_*d*_ < 900 °C. Samples M_5, M_6 and M_7 were prepared following this indication for different combinations of *HR* and *t*. These samples resulted fragmented once extracted from the SS sheath, most probably because of the excessive stress exerted by the SS-crucible walls on the reacted MgB_2_ bulk sample during the post-reaction quench. However, it was still possible to characterize them. We did not investigate *H*_*C2*_ in an extrapolated high-*T*_*d*_ region above 1050 °C, which according to the DoE could lead to *µ*_*0*_*H*_*C2*_ ~ 27 T. Temperatures of ~ 1050 °C represent an upper limit for the mechanical strength of the 2-mm-thick-wall SS crucible, which has to withstand the internal overpressure from the Mg vapors. *H*_*C2*_ values of samples M_1, M_2 and M_3 reproduce the RSM-DoE expectations with a precision within ~ 10%. Discrepancies up to ~ 25% are found for the three samples (M_5, M_6 and M_7) prepared at very high pressure, out of the DoE synthesis-parameter space. Synthesis conditions of M_4 vary from those of M_3 only for the dwell time (50 min in the place of 90 min). However, this sample showed a lower *µ*_*0*_*H*_*C2*_ (by ~ 3.5 T) and a higher *T*_*C*_ (by ~ 1.4 K) with respect to M_3, indicating that the dwell time plays an important role in enhancing the C-doping efficiency at low *T*_*d*_ < 900 °C.

**Table 8:** Range of variation of the synthesis parameters for the Response-Surface-Methodology DoE.

Factors	Coded Units				
	α−	−	0	+	α+
*T* [°C]	850	900	950	1000	1050
*HR* [°C/min]	100	400	700	1000	1300
*t* [min]	20	40	60	80	100
*P* [MPa]	125	187.5	250	321.5	375

**Table 9:** Synthesis conditions (in coded units) of all the samples prepared for the Response-Surface-Methodology DoE. Coded units refer to Table 8.

Sample ID	Randomized order	*T *(coded units)	*H *(coded units)	*t *(coded units)	*P *(coded units)
RSM_1	0	0	0	0	0
RSM_2	2	−	−	−	+
RSM_3	5	−	+	−	−
RSM_4	20	0	α_+_	0	0
RSM_5	17	α_−_	0	0	0
RSM_6	14	+	+	−	+
RSM_7	0	0	0	0	0
RSM_8	12	+	−	+	+
RSM_9	7	−	+	+	−
RSM_10	19	0	α_−_	0	0
RSM_11	3	−	−	+	−
RSM_12	15	+	+	+	−
RSM_13	10	+	−	−	+
RSM_14	21	0	0	α_−_	0
RSM_15	18	α_+_	0	0	0
RSM_16	6	−	+	−	+
RSM_17	24	0	0	0	α_+_
RSM_18	9	+	−	−	−
RSM_19	8	−	+	+	+
RSM_20	11	+	−	+	−
RSM_21	4	−	−	+	+
RSM_22	22	0	0	α_+_	0
RSM_23	1	−	−	−	−
RSM_24	16	+	+	+	+
RSM_25	23	0	0	0	α_−_
RSM_26	13	+	+	−	−

**Table 10:** Synthesis conditions (in coded units) for the 7 samples of the post-RSM-DoE batch. Coded units refer to Table 8.

Sample ID	*T *(coded unit)	*HR *(coded unit)	*t *(coded unit)	*P *(coded unit)
M_1	+	0	0.9 α_+_	α_−_
M_2	+	−	0.9 α_+_	α_−_
M_3	α_−_	+	0.9 α_+_	α_+_
M_4	α_−_	+	0.5 α_+_	α_+_
M_5	α_−_	+	0.9 α_+_	2α_+_
M_6	α_−_	+	1.2 α_+_	2α_+_
M_7	α_−_	0	0.9 α_+_	2α_+_

### In-field electrical transport characterization

**Figure 8:**
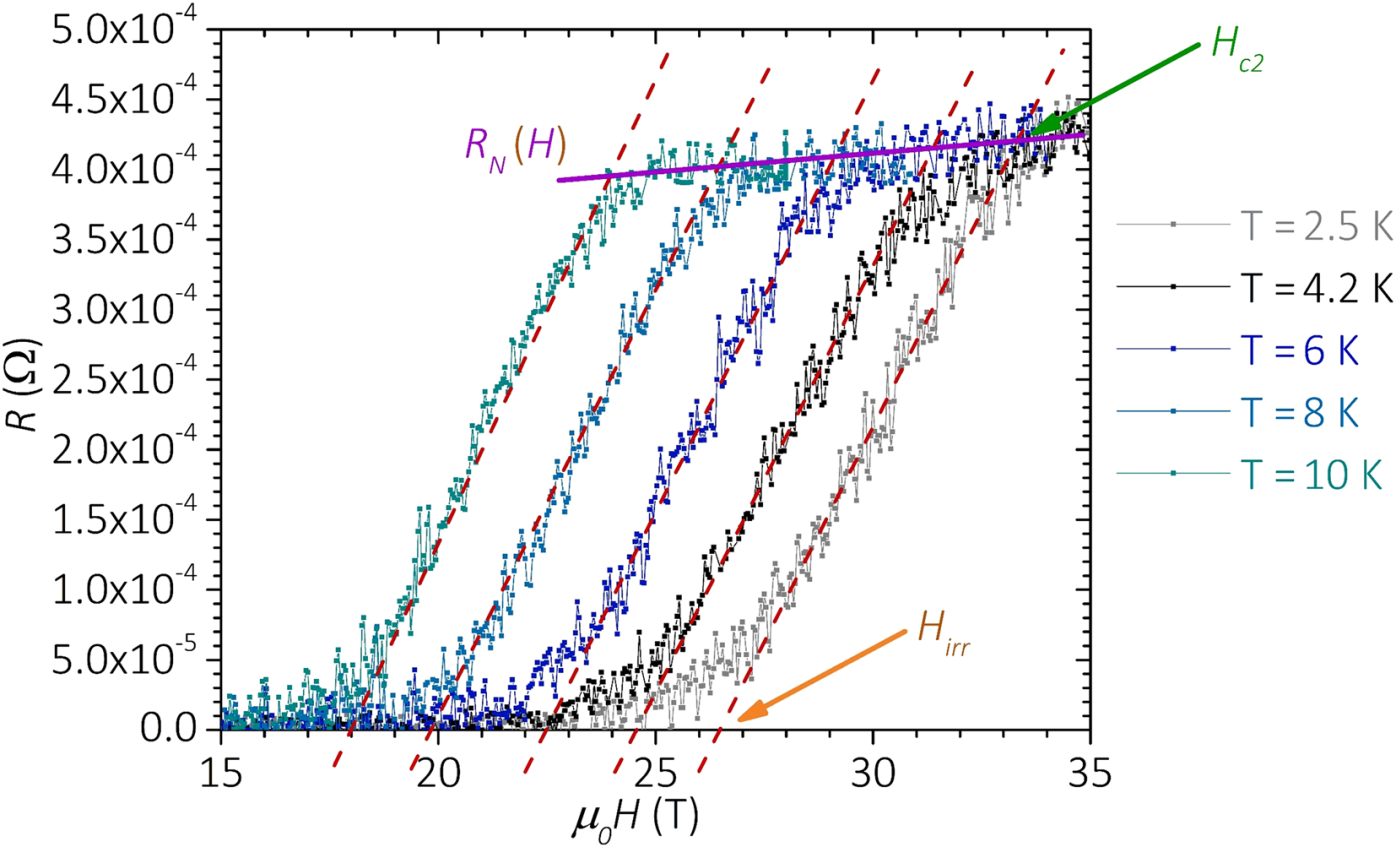
Resistance as a function of the magnetic field for fixed temperatures in the range 2.5–10 K. *H*_*Irr*_(*T*) and *H*_*C2*_(*T*) are defined as the magnetic field values corresponding to the intersection of the linear fit of the superconducting transition (dashed lines) with the superconducting −*R*=0− and the normal-state resistance −*R*_*N*_(*H*)− lines, respectively.

We investigated the samples’ electrical resistance (*R*) as a function of *T* and *H* by standard 4-wire measurements. Most of the samples were tested at the University of Geneva using a laboratory-made low-noise probe^60,61^. We also designed and commissioned at the University of Geneva a dedicated low-noise probe to fit the 35 T magnet bore of the LNCMI facility in Grenoble. Both probes allow measuring up to 4 samples at the same time and choosing the samples’ orientation with respect to the *H* direction. Each sample was powered with excitation current in the range 1–10 mA in order to avoid heating effects. The voltage drop was amplified to increase the signal-to-noise ratio and measured with a nanovoltmeter. To determine the field dependence of the electrical resistance *R*(*H*), we swept the field at a constant rate of ~ 1 T/min. The probing current density was ~ 5 × 10^–2^ A/cm^2^. The *R*(*H*) dependence was investigated for fixed *T* values stabilized with a precision of ± 10 mK. The *R*(*H*) curves, as measured at different temperatures in the sample RSM_17, are reported in Fig. 8 for the sake of clarity. *H*_*C2*_ and *H*_*Irr*_ were evaluated from the intersection of the linear fit of the superconducting transition with the normal-state *R*_*N*_(*H*) and the *R* = 0 lines, respectively. The sample critical temperature (*T*_*C*_) was evaluated from the *R*(*T*) curves acquired at *H* = 0. *T*_*C*_ is defined as the temperature at which the derivative *dR/dT* has a maximum. The width of the superconducting transition is defined as *ΔT*_*C*_ = *T*_*90%*_–*T*_*10%*_, where *T*_*90%*_ and *T*_*10%*_ are the temperatures at which *R*(*T*) is 90% and 10% of the normal state value just above the onset of the superconducting transition, respectively.

### Microstructural and electronic characterization

The samples’ microstructural properties were investigated by X-ray diffraction (XRD) and scanning electron microscopy (SEM) measurements. XRD patterns were collected on the PANalytical Empyrean powder diffractometer with the Bragg–Brentano geometry using the Cu Kα1 monochromatic radiation in the 2θ range between 20° and 120°. We performed a Rietveld refinement on the X-ray patterns by means of the FullProf Suite^62^ in order to evaluate the MgB_2_ lattice parameters (*a*, *c*).

XPS measurements were performed using a Physical Electronics VersaProbe III system with a hemispherical analyser and monochromated Al Kα source. The energy scale linearity was calibrated with Au4f7/2 at 83.86 eV and Cu2p3/2 932.59 eV and data were referenced to the Ag3d5/2 peak at 368.36 eV. All data were measured at room temperature with a pass energy of 55 eV, at a take of angle of 45° and angular acceptance angle of +/− 20°. The samples were electrically grounded during measurement. The X-ray beam size on the sample was ~ 100 µm with a power of 25 W and chamber pressure was less than 1 × 10^–8^ mbar. All samples were polished with sandpaper to remove a surface layer of at least ~ 200 μm in order to remove the layer of material at the surface resulting from spark-erosion cutting. Samples were sputter cleaned in-situ with 2 kV Argon ions for 18 min. Consistently with previous studies, we verified that sputter cleaning duration did not alter significantly the binding energy or FWHM of the MgB_2_ B 1s peak^50^. Sample measurement order was randomized and measurements performed on multiple sample positions over multiple experimental runs produced consistent results.
